# Systems serology: profiling vaccine induced humoral immunity against HIV

**DOI:** 10.1186/s12977-017-0380-3

**Published:** 2017-12-21

**Authors:** Amy W. Chung, Galit Alter

**Affiliations:** 1 Department of Microbiology and Immunology, Peter Doherty Institute for Infection and Immunity, University of Melbourne, 792 Elizabeth St., Melbourne, VIC 3000 Australia; 2 Ragon Institute of MGH, MIT and Harvard University, 400 Technology Square, Cambridge, MA 02139 USA

**Keywords:** Antibody, Systems serology, ADCC, IgG, Subclass, HIV, Vaccine, Fc effector function

## Abstract

The results of the RV144 HIV vaccine, in combination with several recent non-human primate vaccine studies continue to highlight the potentially protective role of non-neutralizing Fc functional antibodies in HIV vaccine design. For many currently licensed vaccines, assays that detect antigen-specific antibody titers or neutralization levels have been used as a correlate of protection. However, antibodies can confer protection through multiple other mechanisms beyond neutralization, or mechanisms which are not dependent on total antibody titers. Alternative strategies that allow us to further understand the precise mechanisms by which antibodies confer protection against HIV and other infectious pathogens is vitally important for the development of future vaccines. Systems serology aims to comprehensively survey a diverse array of antibody features and functions, in order to simultaneously examine the mechanisms behind and distinguish the most important antibody features required for protection, thus identifying key targets for future experimental vaccine testing. This review will focus on the technical aspects required for the application of Systems serology and summarizes the recent advances provided by application of this systemic analytical approach.

## Introduction: Vaccine-induced humoral immunity

Vaccines are one of the world’s most powerful public health interventions. Many of the currently licensed human vaccines are believed to confer protection through the induction of antigen-specific antibodies [1]. These include vaccines against a range of viral pathogens such as hepatitis [2], human papilloma virus [3], polio [4], influenza [5] and yellow fever [6], and the bacterial pathogens including pneumococcus [7] and pertussis [8].

The standard method of assessing vaccine-induced protective humoral immunity is through assays that detect either the level of antigen-specific antibody titres post vaccination, (typically via enzyme linked immunosorbent assays (ELISAs)), the quantity of ‘functional’ antibody responses through inhibition of the pathogen or toxin (e.g. neutralization for polio virus, hemagglutination assay (HAI) for influenza) or opsonophagocytic activity (e.g. pneumococcal bacteria) [1, 9]. These conventional immunological methods are extremely valuable, as they are high throughput, easily standardizable, and thus will remain essential for evaluating vaccine responses. However, the caveat to many of these assays is that they measure a single or very limited number of humoral characteristics, and it is likely that other antibody-mediated mechanisms also contribute to protection against infection in vivo. For many infectious diseases, protection from or infection control is often observable in the absence of antibody neutralization. Instead, accumulating evidence across a spectrum of vaccines has suggested a critical role for additional, non-neutralizing, antibody functions, that is the ability of an antibody to interact with other immune components and effector cells via their Fc portions to mediate killing or control of the pathogen. These mechanism include, but are not limited to, antibody dependent cellular cytotoxicity (ADCC), antibody dependent cellular phagocytosis (ADCP), antibody dependent complement deposition in both protection from and control of HIV [10–13], influenza [14, 15], HSV [16, 17], Ebola virus [18], and malaria [19–21]. Thus, alternative strategies that allow us to further understand and assess the precise mechanisms by which antibodies confer protection against pathogens is vitally important for the development and testing of future vaccines.

## HIV vaccines

Three decades after the discovery of HIV, the journey towards the development of a protective HIV vaccine is still ongoing. While the overarching goal of an HIV antibody-based vaccine is to induce broadly neutralizing antibodies, this historically has not proven to be an easy feat [22]. To date, there have only been four different HIV vaccine concepts that have been tested in a total of six human efficacy trials [23]. Of these, the RV144 ‘Thai’ Phase III trial was the first, and only, human HIV vaccine trial to demonstrate moderate efficacy (31.2%) [24]. Surprisingly, this vaccine did not induce broadly neutralizing antibody responses. Instead non-neutralizing antibodies that recognize a specific region of the HIV Envelope (ENV) variable region 1 and 2 (V1V2) were associated with reduced risk of infection [25, 26]. Additional follow up analysis of the RV144 trial revealed that ADCC, antibody avidity to ENV and low tier neutralizing antibodies in the absence of IgA were secondary correlates of reduced risk of infection [25].

Based on these discoveries, a major focus of the HIV antibody vaccine field has been dedicated to understanding the functional role of these HIV-binding non-neutralizing antibodies [27]. This has resulted in significant advancements in the technologies available to interrogate non-neutralizing functional antibody responses [28]. It has also emphasized that assessing vaccine-induced humoral immune responses by quantifying the level of antigen-specific antibody titres alone is not a sufficient predictor of antibody protection or even Fc-effector functional capacity. Indeed, studies comparing the moderately protective RV144 vaccine trial [24], consisting of ALVAC-HIV (vCP1521) prime and AIDSVAX B/E protein boosts, with its non-efficacious predecessor, VAX003 vaccine [29], consisting of AIDSVAX B/E protein, revealed that VAX003 resulted in significantly higher total HIV-specific IgG levels compared to RV144 [30, 31]. However, the elevated levels of HIV-specific IgG induced by VAX003 were of a less functional nature, with elevated levels of poorly functional IgG4 subclass antibodies, that competed for antigen occupancy, thereby blocking Fc effector functions [31]. In comparison, RV144 vaccination induced lower total HIV-specific IgG titers, but resulted in enhanced levels of highly functional IgG3 subclass antibodies that had the capacity to induce polyfunctional non-neutralizing responses including ADCC, ADCP and antibody-mediated activation of NK cells [31]. These studies highlight the complexity of antibody interactions in vivo and suggest that broader Fc effector profiling and analytical methods may be required to objectively and comprehensively dissect vaccine induced Fc effector functions, in order to identify protective antibody immune features.

## Hierarchical layers of antibody features modulate Fc effector functions

Upon vaccination, humoral diversification forms the foundation of the humoral immune response, that aims to target foreign antigen as broadly and effectively as possible [32]. This response gives rise to polyclonal antibodies that are tuned by genetic, molecular and environmental factors to selectively respond in a pathogen-specific manner. The combined activity of these polyclonal pools is determined by a cumulative framework of antibody features; specificities dictated by the antibody Fragment antigen binding (Fab) region and Fc receptor binding capacity directed by the antibody Fragment crystalizable (Fc) region [33]. For example, a subset of vaccine-induced antibodies may work in a cohesive, collaborative fashion, forming high avidity immune complexes with a preference towards activating Fcγ Receptor engagement and hence exhibiting a more functional Fc effector profile. However, a vaccine might also simultaneously induce antibodies with competing features, potentially blocking immune complex formation or reducing Fcγ Receptor engagement, thus potentially hindering Fc effector functions. Using the earlier described non-protective VAX003 vaccine as an example: VAX003 vaccination induced high levels of total HIV-specific IgG antibodies, however with proportionately elevated levels of weakly functional HIV-specific IgG4, that competed for antigen occupancy with more functional HIV-specific antibody subclasses (e.g. IgG1 and IgG3), overall resulting in a less functional immune response [30, 31]. However, depletion of IgG4 antibodies from IgG samples, resulted in significantly higher functional antibody responses [31]. Similarly, depletion of the more functional HIV-specific IgG3 from vaccine samples resulted in a decrease in function. Thus collectively, functional antibody responses are the result of a dynamic equilibrium of the combination of these enhancing and competitive inhibitory antibody profiles, and the immune environment in which these antibodies are placed. Systems serology therefore aims to comprehensively examine as many of these antibody features as possible, in order to assess the mechanisms behind specific Fc effector responses and identify the most important antibody features required for superior Fc functional activity or protection induced by vaccination, pinpointing the key targets for future experimental validation and additional quantitative models.

## Systems serology

Systems tools have revolutionized our understanding of basic immune processes including immune responses to vaccination, disease and immune development. Key to this developing area of research, is the comprehensive and unbiased collection of data to capture as much information as possible to inform and identify previously unappreciated processes in biology. This field has lead to the development of many OMIC tools including genomics (the whole molecular code) [34, 35], transcriptomics (the whole transcriptional code) [36], proteomics (the building blocks and communicators within the system) [37] and metabolomics (the nutritional/activity state of the system) [38]. While all these tools provided deep information at the cellular level, they provided little resolution related to the humoral immune response, that is composed of polyclonal antibodies that can work together to modulate a range of different anti-pathogenic functions. Thus Systems serology was designed to address this area, with the specific aim to capture and assimilate as much information as possible related to the modulation of functional humoral immune responses.

Systems serology consists of a fusion of high throughput experimental techniques aimed at dissecting the antibody features and functions, followed by a range of computational methods to help mine through and provide an unprecedented depth of understanding to the profiled antibodies. Simplistically, the experimental assays used to interrogate antibodies can be divided into (1**)** *Biophysical assays* (Summarized in Table 1) and (2) *Functional assays* (Summarized in Table 2). *Biophysical assays* reflect the immediate genetic and molecular constraints of vaccine-induced antibodies that dictate antigen recognition and Fcγ Receptor engagement. In parallel, *functional assays* are used to examine, in vitro, the functional potential of these antibodies, which is dependent upon the availability of specific effector cells or immune environments. Upon compilation of these extensive datasets, both supervised and unsupervised machine learning *computational techniques*, commonly used in Systems biology, can be applied to allow for the examination of the multiple layers of antibody information, in order to help decipher and identify important features associated with protection and/or Fc functional activity.

**Table 1:** Biophysical antibody profiles

	
Fragment	Fab			Fc				
Feature	Antigen target	Epitope target	Antigen affinity	Isotype	Subclass	Glycans	FcR/C’ binding	FcR affinity
Examples of measurements	gp120 gp41 p24	Scaffolds -V1V2 -SOSIP -linear peptides	Dissociation constant (k-off)	IgA IgM IgG	IgG1 IgG2 IgG3 IgG4 IgA1 IgA2	~30 glycan combinations Eg. Fucose, bisecting GLNAc, Galactose, Sialic Acid	C1q MBL FcγRI FcγRIIa FcγRIIb FcγRIIIa FcγRIIIb	Equilibrium constants (KD)
Examples of Assays	Multiplex ELISAs	Multiplex ELISAs ICS	SPR Chaotrope	Multiplex ELISAs	Multiplex ELISAs	Mass Spec HPLC CE Multiplex	ELISA Multiplex	SPR
References	[104]	[31, 40]	[105]	[104]	[104]	[53, 87, 106]	[44, 87]	[105, 107]

**Table 2:** Profiling functional Fc effector functions

	
Effector	Complement activation	ADCP	ADCC	Antibody dependent Cytokine secretion	Antibody dependent Chemokine secretion	Antibody dependent Virus Inhibition
NK cells	−	−	+	+	+	+
Neutrophils	−	+	+	+	+	ND
Monocytes	−	+	+	+	+	+
Dendritic cells	−	+	−	+	+	ND
Complement	+	ND	ND	ND	ND	+
Examples of Assays	Complement assay ELISA	Bead assay Virion assay	Cr51 Release RFADCC GranToxiLux Luciferase ADCC BVADCC	ICS	ICS	ADCVI
References	[86, 108]	[10, 62, 63, 109–111]	[28, 112–117]	[31, 118]	[31]	[13]

### Biophysical antibody features in HIV vaccines and infection

#### Antibody epitope recognition

Vaccine-induced humoral immune responses can produce a multitude of polyclonal antibodies, that have the potential to target an extensive array of conformational and linear epitopes, which can be detected by multiple methods (Table 1—left columns). In the case of HIV vaccination, the required epitopes can be even more complex, as sequence variation across different strains and clades can alter not only epitope recognition but also ENV protein glycosylation patterns [39, 40]. In this context, the uniqueness and strength of functional Fc-mediated antibodies lies in their ability to target a large array of epitopes, beyond the few limited neutralizing antibody sites [41], thus potentially allowing for the exploitation of alternative conserved sequences as epitopes [40, 42]. It is also due to the ability of non-neutralizing antibodies to target a diverse array of epitopes, that functional antibodies may potentially have greater breadth of recognition across different HIV clades [43, 44], an important measure to assess, due to the extreme diversity of HIV strains. Of interest, ADCC activity has also been observed against various other highly conserved HIV viral antigens including gag, pol and the accessory proteins, and has been linked to slower disease progression [45–47], however, due to the intracellular location of many of these epitopes, their relevance to protection from infection is still unclear. In addition to the nature of the epitope recognized, both the strength of antibody engagement with the antigen [48] and epitope availability/masking can impact immune complex formation [49]. Together, it can be hypothesized that higher affinity antibodies, recognizing readily available/conserved epitopes, will form prolonged immune complexes and hence have increased opportunities for Fcγ receptor engagement.

#### Antibody Fc structure

Despite its name, an antibody’s Fragment crystalizable (Fc), or constant region can also be highly modulated [33]. These changes can influence an antibody’s ability to bind and engage Fc receptors present on innate immune cells or other immune components, such as complement. Binding ability can be modulated through small biophysical differences ranging from isotypes (e.g. IgG, IgA, IgM, IgE, IgD) or subclasses (e.g. IgG1-4) [50], to single amino acid polymorphisms [51], small glycan modifications [52, 53] and immune complex size [54], that collectively can contribute to differences in Fc–FcR binding ability. Several high throughput standardized assays have been developed to allow for the in-depth assessment for these features (Summarized in Table 1- right columns). In the context of HIV, multiple studies have demonstrated the importance of subclass, isotype and glycosylation state for control of disease and/or protection [30, 31, 55–57]. For example, the results of the RV144 immune correlates analysis has suggested that serum IgA levels may be detrimental to vaccine efficacy [25], potentially through competition of more functional vaccine-induced IgG, capable of mediated ADCC [58]. However, in contrast, results from a non-human primate passive antibody immunization study, demonstrate that dimeric IgA1 was superior to both IgA2 and IgG1 in providing protection against SHIV mucosal infection [59]. Overall these data suggest that IgA may have both protective and detrimental effects on HIV immunity, dependent on its location.

#### Antibody dependent functional responses

Prior to the results of the RV144 trial, the majority of functional Fc-mediated antibody research focused upon ADCC responses through assays that detected the lysis of target cells or inhibition of viral replication [13, 60]. Many of the assays were highly complex, difficult to standardize, and time consuming, requiring the use of radioactive cells such as the chromium release assay or virus-infected primary target cells [61]. However in the past decade, several high throughput ADCC and other functional Fc-mediated assays have been developed, allowing us to probe multiple other non-neutralizing antibody functions (Table 2). These include, but are not limited to, antibody dependent complement dependent cytotoxicity (ADCDC) [55], antibody dependent cellular (or viral) phagocytosis (ADCP) [62, 63], antibody mediated effector cell cytokine and chemokine secretion [64] along with antibody dependent cellular viral inhibition (ADCVI) [13]. Antibodies also have the capacity to induce multiple other effector functions including, but not limited to antibody dependent enzymatic release/respiratory burst [21] and antibody mediated mucus trapping [65]; however, high throughput qualitative robust assays to assess these functions in HIV are still in development.

#### Fcγ receptor and effector cell diversity

Fc-functional immune responses are often dictated by the innate immune effector cell that is being activated and its respective FcγR repertoire. It is for that reason that earlier ADCC studies commonly examined NK cell-specific antibody function, due to the fact that NK cells primarily express a single activating FcγRIIIa (CD16a) allowing for easier interpretation of results [66]. However, three distinct classes of FcγRs are expressed on human innate immune cells, with varying levels of expression dependent on cell type. These include FcγRI, FcγIIa/b/c, and FcγIIIa/b, which bind the different IgG subclasses with varying proficiency, and can cause either activation or inhibition of the effector cell [50]. Furthermore, a range of FcγR polymorphisms have been identified in humans, some of which have greater Fc binding affinity and hence are associated with enhanced Fc effector functional capacity. For example, individuals carrying the high affinity FcγRIIa H131 polymorphism, most commonly associated with enhanced ADCP, have positive outcomes in both cancer [67] and infectious disease studies, including HIV [68, 69]. In contrast, the FcγRIIIa V158 polymorphism, associated with enhanced ADCC functionality, has been linked with better outcomes within the monoclonal therapeutics cancer field [70, 71]. Conversely, however, this same polymorphism has been associated with HIV disease progression [72] and the lack of protection from the VAX004 vaccine trial [73]. Thus assays able to capture the functional activity of antibodies through multiple FcγRs may provide enhanced resolution of the vast repertoire of functions that may be leveraged to control/kill HIV, while assays utilizing individual FcγRs provide an opportunity to critically capture a quantitative view of a specific antibody function. Furthermore, FcγR expression profiles may change with infection status and site of infection [74], all which must be considered in the process of establishing meaningful assays for the analysis of functional humoral immune responses, as these FcγRs compete on the surface of innate immune cells for immune complex binding. For many of the described Fc effector assays, specific FcγR bearing innate immune cells can be isolated and their FcγR polymorphisms and expression levels can be determined, so that the Fc-mediated immune responses can examined and later reconstituted in a biologically meaningful manner.

#### Assay selection and technical considerations

As with all experimental assays, standardization and optimization is critical to enable accurate cross-study comparisons. Control samples are essential for validation and quality control. Furthermore, sample and control replicates should be completed for all assays to confirm assay reliability, and data accuracy. Data analysis techniques can be utilized to confirm assay and data quality, these include determining signal to noise ratios or calculating the Z score [75].

It is important to note that a more robust, optimized assay does not necessarily reflect biological relevance. For example many of the most reproducible functional antibody assays utilize cell lines that are representative of functional immune responses [62, 76]. There are multiple advantages to these assays including high throughput data quality and repeatability on an intra-laboratory basis. However it should be acknowledged that they often do not represent the biological diversity as observed when using assays that utilize primary cells [77]. In contrast, assays that involve the isolation and use of primary FcγR receptor cells, especially from mucosal tissues to assess for functional humoral responses, may be highly biologically relevant, however currently, robust high throughput technologies with the capacity to assess these responses are less developed. Thus assay selection may also take into consideration current assay technology constraints and biological significance.

The full spectrum of biophysical and functional assays should ideally be collected from all samples, thus allowing for a sweeping assessment and the application of computational approaches to the entire cohort. While admittedly it is not always feasible or practical to collect entire data sets from large cohorts, estimating smaller sample sizes for reliable analysis should be carefully considered prior to experimental data collection, as this can be influenced by multiple factors, including the computational methods applied and dimensionality of the data (e.g. number antibody features and functions) [78].

#### Computational techniques

When all measurements are compiled, systems-level computational analysis can then be applied to probe for mechanistic insights or antibody biomarkers of protection. A variety of different “*machine learning*” or data-driven computational approaches have been previously applied to Systems biology and Systems vaccinology (reviewed [79, 80]) and more recently Systems serology [81]. These approaches are broadly classified as ‘*unsupervised*’ and ‘*supervised*’ approaches. ‘*Unsupervised*’ approaches explore the compiled datasets, without predefining groups to base the analysis upon, thus allowing for the identification of natural similarities or “clusters” within the data. A major advantage of unsupervised systemic examination of broad Fc effector profiles, is the ability to comprehensively explore the mechanisms behind specific functions or correlates of protection in an unbiased fashion, potentially revealing novel linkages that would not normally be identified by traditional approaches. Previous unsupervised approaches that have been successfully applied in Systems serology research include principal component analysis (PCA) and correlation networks [82–84].

In contrast, ‘*supervised*’ approaches include the addition of predefined groups or classifications, from which the analysis is based, with the goal of identifying key parameters associated with the predefined groups or “classes”. In the context of Systems serology, these groups may be related to a specific advantageous Fc effector function (e.g. ADCC or ADCP etc.) or clinical outcome (e.g. protective versus non-protective vaccine), with the aim to identify novel correlates of protection or key antibody features associated with Fc effector functions. Supervised analytical techniques that have previously been applied to Systems serology include partial least squares discriminant analysis (PLSDA) and decision tree analyses [82, 84–86].

In addition, current high throughput assays used to measure antibody structure and function can generate tens and more often hundreds of data points per sample (for example, Fc/isotype multiplex arrays or peptide epitope mapping) [87]. The strength of this comprehensive approach is that all samples are assessed in an unbiased manner for as many functional antibody features as possible. The downside of this analysis is that many of these antibody features and functions have the potential to be highly correlated with each other, thus highlighting multiple features and often resulting in over complex models. This can create overwhelmingly large data sets, where the total number of antibody measurements can outweigh the number of samples tested. Thus feature selection methods are useful for minimizing the number of selected features, correcting for the effect of over fitting data, which can improve model performance and help reduce complexity of the data (reviewed in [88, 89]).

Overall, application of these machine learning computational techniques in Systems serology research, has the ability to highlight the most critical associations between antibody features and functions with protection from HIV infection, thus becoming a highly targeted and comprehensive hypothesis driving tool that can accelerate our understanding of the humoral immune system, and help to identify key areas of interest for further experimentation, vaccine testing or exploration by additional quantitative models.

### Systems approaches for the analysis of functional antibody responses in human vaccination

Previous application of Systems biology and vaccinology approaches has resulted in the identification of genetic or immune signatures that correlate with vaccine efficacy for yellow fever [90], influenza [91] and malaria [92, 93]. These predictive signatures can be used to identify novel mechanistic insights and aid the development of future vaccine strategies, while also serving as valuable biomarkers to screen for vaccine efficacy in future trials.

In our application of Systems serology to different human HIV vaccines [84], we utilized a variety of modelling and data clustering techniques to examine the antibody profiles of the moderately protective RV144 trial (ALVAC and AIDSVAX B/E [24]) and two non-efficacious HIV vaccine trials: VAX003 (AIDSVAX B/E [29]); and HVTN204 (DNA/rAD5 [94]). Importantly, application of both unsupervised and supervised analysis approaches were able to differentiate between AIDSVAX B/E protein containing vaccines (i.e. RV144 and VAX003) and DNA/rAD5 vaccination (HVTN204). Furthermore, in a supervised analysis that applied LASSO [95] (least absolute feature shrinkage and selection operator) for feature selection followed by PLSDA [96] (partial least-squares discriminant analysis), we were able to clearly differentiate between RV144 and the non-efficacious VAX003 trial samples, reconfirming previous studies that associated RV144 with enhanced levels of IgG3 and VAX003 with an elevated IgG4 signature [30, 31].

A similar supervised LASSO and PLSDA approach was applied to RV144 recipients separated into those with high or low V1V2 responses, as a representation of low versus high risk of infection, based on the original immune correlate analysis [25]. High IgG V1V2 responder profiles, with predicted low risk of infection, were associated with high levels of HIV-specific IgG1 along with multiple or potentially “polyfunctional” Fc effector functions including ADCP, ADCC, and antibody dependent NK cell activation. Follow up studies now also suggest that high V1V2 responders not only have enhanced Fc functionality, but may also have increased ADCC epitope breadth [44]. In contrast, low IgG V1V2 responders, predicted to have a higher risk of infection, were associated with HIV-specific IgA, recapitulating earlier immune correlate studies [25]. Of interest, repetition of this analysis comparing high and low IgG3-V1V2 responders (which was also detected to be a correlate of protection [30]), associated high IgG3-V1V2 responders with activating FcγRIIa, required for ADCP, and FcγRIIIa, which is critical for NK cell activation and ADCC. These results reinforce the importance of polyfunctional Fc-effector functions. However, contrary to the total IgG-V1V2 analysis, IgA was not identified as a correlate of the low IgG3 V1V2 responder profile, suggesting that IgA responses may exert varying degrees of modulation/interference with Fc effector responses. Further studies to dissect the divergent roles of IgA, potentially in a V1V2 subclass specific manner, may be warranted.

Functional humoral immune responses are induced through the engagement of immune complexes that consist of varying antibody composition, antigen specificity and FcγR engagement. Thus the interactions required for Fc functions are an ideal model for the application of network and pathway analyses that are commonly applied in bioinformatics analyses (reviewed in [97, 98]). These approaches are useful as they allows us to simultaneously examine the connectivity between different antibody functions and features to examine mechanisms behind antibody functional activity, in an unbiased, objective manner. These interactions are often displayed as networks that illustrate the degree of connectivity between different features. Examination of the structural organization and topology of these biological networks can provide us with information behind key interactions that regulate a specific functional immune response. Application of correlation networks analysis to the different vaccine trials revealed extremely different network topographies or ‘humoral fingerprints’. Importantly the connectivity analysis using RV144 samples reiterated earlier PLSDA results, demonstrating that ENV-specific IgG1 and IgG3 responses were interconnected with multiple antibody effector functions including ADCC, ADCP and ADCDC, which was not observed for the other non-efficacious vaccine trials.

## Systems serology approaches in non-human primate models efficacy trials

Similar Systems serology approaches have also been applied to several SIV and SHIV vaccine trials [86, 99, 100]. A combination of Systems biology and Systems serology analyses was applied to a non-human primate (NHP) RV144-like vaccine and challenge study consisting of an ALVAC prime followed gp120 protein boost vaccine strategy, comparing the adjuvants Alum and MF-59. Systems analysis of this study was able to associate mucosal innate lymphoid cells producing IL-17, RAS activation, and mucosal V2 antibodies with delayed acquisition of infection [100]. A second NHP vaccine study, consisting of Ad26 prime followed by ENV protein boost, applied similar systems serology techniques and associated the observed 50% protection from repeated SIV challenges with polyfunctional Fc effector functions including ADCP, ADCC, ADCD and antibody mediated NK cell activation [86]. Collectively, these NHP systems serology studies reinforce the results of the previous RV144 immune correlates analysis [25] and in combination with the RV144 Systems serology study [84], suggest that future HIV vaccines capable of eliciting polyfunctional Fc effector profiles, with a preferential upregulation of IgG3 subclasses and high levels of V2 antibodies may be able to reduce the risk of HIV infection.

In the future, additional Systems serology analysis of vaccine trials in NHP, with known efficacy will provide us additional opportunities to identify correlates of protection. Importantly, NHP vaccine trials provide us with the unique opportunity to dissect responses to a deeper degree, often not possible in human vaccination, both by way of sample collection and information about the challenge virus [101]. While there are significant differences in both FcγR and IgG biology between humans and NHP, dissecting the NHP FcγR and Immunoglobulin H locus is an area of growing intense research [102]. As our understanding of this field grows, we may be able to better build parallels between NHP and human protective responses that will allow us to build predictive models that may help guide the HIV vaccine field.

## Systems serology: A constantly expanding platform

It is important to note that the technologies available to measure biophysical antibody features and functions are constantly evolving to allow for the assessment of more diverse cell types, cellular environments, receptors and antigen specificities. No doubt, in the future, many other high throughput assays will be developed to allow for advanced measurements of antibody features. Next-generation sequencing and bioinformatics have already enabled probing of the antibody repertoire and greater understanding of the evolution of broadly neutralizing antibodies, and these techniques could be modified and applied to explore the evolution of polyfunctional non-neutralizing or V1V2 antibodies [103]. Similarly, new technologies are being developed to better examine mucosal antibody features, as well as functional assays that better represent in vivo Fc-functional responses from different effector sites such as blood, gut, and reproductive mucosal environments. Additionally, a multitude of alternative analytical approaches could be applied to qualitatively evaluate vaccines responses. Application of Systems serology is not limited to HIV vaccinology, but could potentially be applied to numerous other infectious and autoimmune diseases [83], or could be used to examine differences in humoral immune responses amongst individuals of differing gender, age or ethnicity. There is no doubt that Systems serology is still in its early developmental stages and will continue to evolve over time to capture broader Fc-effector profiling and improved multi-dimensional analytic approaches, providing us with an increasingly comprehensive understanding of the humoral immune response required for the identification of the most desirable vaccine profiles in order to guide the rational design of future HIV vaccine candidates.
